# Duplication of the *ALDH1A2* gene in association with pentalogy of Cantrell: a case report

**DOI:** 10.1186/1752-1947-7-287

**Published:** 2013-12-30

**Authors:** Matthew B Steiner, Jaime Vengoechea, Ronnie Thomas Collins

**Affiliations:** 1 Division of Pediatric Cardiology, Arkansas Children’s Hospital, Little Rock, USA; 2 Department of Pediatrics, The University of Arkansas for Medical Sciences, Little Rock, USA; 3 Division of Genetics, The University of Arkansas for Medical Sciences, Little Rock, USA

**Keywords:** 15q21.3, *ALDH1A2*, Pentalogy of cantrell, *RALD2*, Retinoic acid

## Abstract

**Introduction:**

The pentalogy of Cantrell is rare clustering of congenital defects, first described by Cantrell and colleagues in 1958. The exact pathogenesis for the pentalogy remains unknown and no specific genetic abnormalities have been correlated; however, a failure of embryogenesis has been suspected. The microduplication of chromosome 15q21.3 (57,529,846 to 58,949,448) found in our patient with pentalogy of Cantrell has not been described previously.

**Case presentation:**

We describe a case of a newborn Caucasian male baby with prenatally diagnosed pentalogy of Cantrell and a novel maternally inherited copy number variant detected by chromosome microarray analysis. Among the genes within the duplicated region is *ALDH1A2*, encoding the enzyme retinaldehyde dehydrogenase type 2.

**Conclusion:**

Vital for retinoic acid synthesis during early development, *ALDH1A2* has previously been demonstrated in animal models to have a strong association with congenital heart disease and diaphragmatic hernia, two key elements comprising pentalogy of Cantrell. It is possible that perturbation of retinoic acid levels during development secondary to this microduplication could underlie the pathology observed in the current case of pentalogy of Cantrell.

## Introduction

In 1958, Cantrell and colleagues described the cases of five patients with a rare clustering of congenital defects, consisting of a midline, supraumbilical abdominal wall defect, a defect of the lower sternum, a deficiency of the anterior diaphragm, a defect in the diaphragmatic pericardium and intracardial defects [1]. We performed a chromosomal microarray analysis in a baby born with pentalogy of Cantrell that demonstrated a novel gain in copy number on an interval of 15q21.3. Among the genes found in the repeated segment was *ALDH1A2*, which encodes the enzyme retinaldehyde dehydrogenase type 2. Retinaldehyde dehydrogenase type 2 is critical for the conversion of vitamin A into all trans-retinoic acid, which is a morphogen that is known in vertebrate and avian models to be integral for normal cardiac development and diaphragm formation in the early stages of gestation. We believe this novel copy number variant in association with pentalogy of Cantrell may be significant and therefore report our experience with this patient.

## Case presentation

A 32-year-old G2P1 Caucasian expectant mother was referred for a fetal echocardiogram at an estimated gestational age of 20 weeks secondary to the presence of a fetal omphalocele. The echocardiogram demonstrated the fetus to have a large omphalocele, a lower sternal defect, and a midline cardiac apex with a small portion of the ventricles protruding through a pericardial and diaphragmatic defect onto the surface of the liver. The intracardiac anatomy was found to be consistent with tetralogy of Fallot. The care of the mother was transferred to a high-risk, maternal-fetal medicine physician with a plan for delivery at a tertiary care center and subsequent transfer to a nearby children’s hospital for postnatal management.

The mother presented in active labor at 38 4/7 weeks’ gestation. Rupture of membranes, with meconium staining, occurred one hour prior to delivery, and a male baby was delivered without complication via a vaginal birth after a Cesarean section. At delivery, the baby demonstrated no respiratory effort, and his heart rate was <100 beats per minute. The baby was placed in a plastic bag and intubated on the first attempt. His heart rate quickly improved. A physical examination demonstrated a sternal defect covered by intact skin and a palpable cardiac impulse just beneath his skin at the level of the sternal cleft. There was a large, epigastric omphalocele containing visible liver, stomach and intestines. The remainder of the physical examination was normal, including a three-vessel umbilical cord. His birth weight was 3630g and Apgar scores were 2, 6 and 7 at 1, 5 and 10 minutes of life, respectively. Our patient was hemodynamically stable and was then transferred to our institution.

In our neonatal intensive care unit, we adopted a conservative management plan of the omphalocele: to forgo surgical repair and undertake daily dressing changes with antiseptic ointment while awaiting spontaneous epithelialization. Transthoracic echocardiography confirmed the presence of tetralogy of Fallot, with mild valvar pulmonary stenosis, and continuous, mildly hypoplastic branch pulmonary arteries. With spontaneous closure of the ductus arteriosus, our patient maintained a normal oxygen saturation level, and we planned for a complete repair on an elective basis at 3 to 6 months of age.

**Figure 1:**
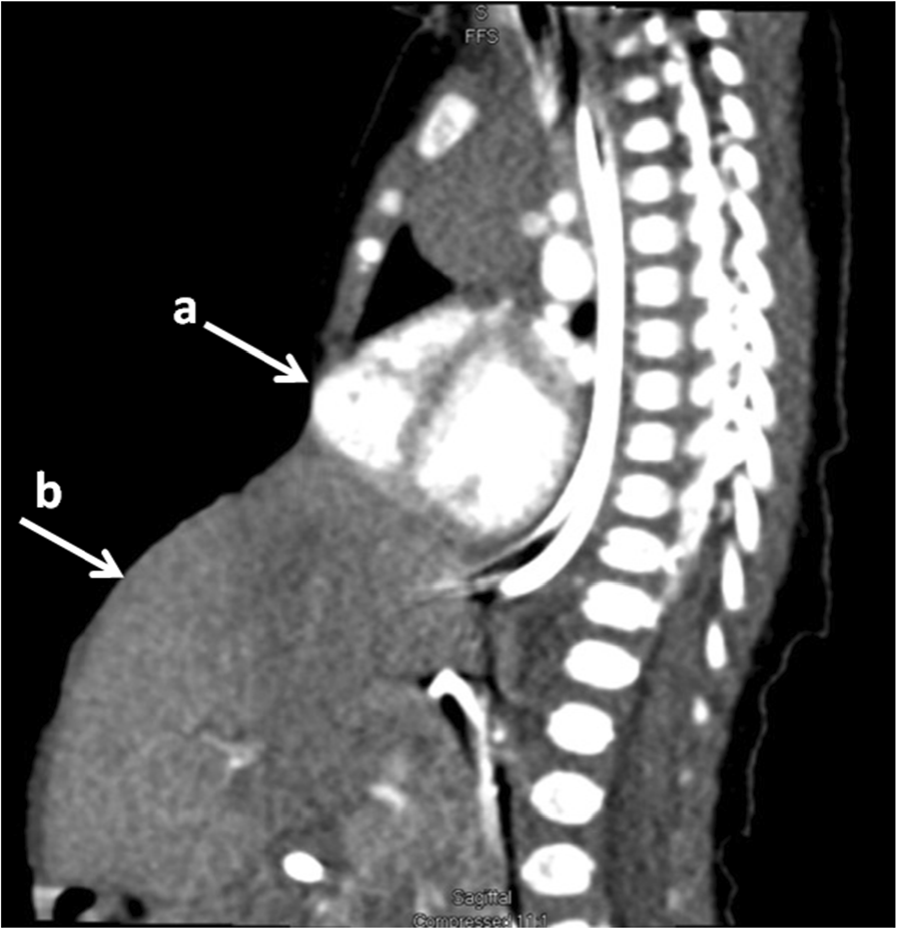
**Computed tomography scan.** A sagittal view from a contrast-enhanced computed tomography study demonstrating protrusion of the right ventricle through the sternal defect **(a)** and extra-abdominal position of the organs in the large omphalocele **(b)**.

A computed tomography scan of his thorax confirmed the deficiency of his lower sternum, with the anterior aspect of his right ventricle just beneath the skin, as well as the deficiency of his anterior diaphragm (Figure 1). His liver, spleen and stomach were herniating through the anterior abdominal wall defect into the omphalocele. We found dependent atelectasis of his lungs. The remainder of the computed tomography scan and head and renal ultrasounds were normal. The baby was extubated on his 20th day of life, and continues to have daily dressing changes to the omphalocele at the time of writing.

A 180K Oligo Chromosome Microarray Analysis, performed by Ambry Genetics (Aliso Viejo, CA, USA) was significant for a gain in copy number (x3) in chromosome 15q21.3 with a minimum interval from 57,529,846 to 58,949,448. Parental fluorescent *in situ* hybridization analysis using probe RP11-345H13 revealed the presence of a 15q21.3 duplication within the mother, indicating maternal inheritance from a phenotypically normal mother with no known diaphragmatic, sternal, pericardial, abdominal wall or intracardiac defects.

## Discussion

Microarray complete genome hybridization allows for high resolution, genome-wide screening for submicroscopic chromosomal imbalances. The technology has allowed screening of the entire genome for copy number variants and has proven useful in detecting imbalances in neonates with birth defects [2] and in patients with congenital heart disease [3,4]. A microarray analysis does not specifically assess if a gain in copy number alters or disrupts the expression of the genes, but partial gene duplications would be anticipated to disrupt gene function [5]. Numerous studies have identified various copy number variants to play a role in structural birth defects, including congenital diaphragmatic hernia and various forms of congenital heart disease [6]. Screening babies with microarray analysis has become routine practice in many institutions in cases of selected congenital birth defects.

The specific region on chromosome 15q21.3 duplicated in our patient is known to contain nine genes: *ADAM10* (partially duplicated at the 3′ end), *ALDH1A2*, *AQP9*, *CGNL1*, *GCOM1*, *GRINL1A*, *LIPC*, *LOC283663* and *TCF12* (partially duplicated at the 5′ end). Only *ADAM10*, *ALDH1A2* and *LIPC* have been reported previously to have clinical significance. The clinical correlations reported for *ADAM10* and *LIPC* have no apparent relevance to our case.

An alteration in *ALDH1A2* may be clinically relevant in our patient. *ALDH1A2* (also known as *RALDH2*) codes for the enzyme retinaldehyde dehydrogenase type 2, which is crucial for the conversion of dietary vitamin A (retinol) to retinoic acid. Retinoic acid is known to be a powerful morphogen important during early development for axial patterning and in later development for organogenesis [7]. Retinoic acid cannot be synthesized *de novo* by animals, and must be obtained from preformed dietary vitamin A, which is then bound to retinol binding protein and transported into the cell by STRA-6, a membrane receptor. Within the cell, retinol is first oxidized to retinaldehyde, which is then further oxidized to retinoic acid. The oxidation to retinoic acid is catalyzed by the retinaldehyde dehydrogenase family of enzymes; of these three, retinaldehyde dehydrogenase type 2 is the major form involved in early embryonic and cardiac development [7].

The concentrations of retinoic acid are tightly regulated during development, and both deficiency and excess result in altered development in animal models [8]. The vertebrate heart is particularly sensitive to variations in retinoic acid signaling. Avian embryos subjected to vitamin A deficiency have been used to model vitamin A effects on very early heart development. The observed phenotypes included a grossly abnormal cardiovascular system. The critical time period for retinoic acid regulation corresponds to the first two to three weeks of human pregnancy [8]. This same embryologic period is associated with failed progression of the lateral mesoderm into the transverse septum of the diaphragm, which has been implicated to underlie pentalogy of Cantrell [1].

When screening 133 patients with congenital heart disease for variations within the *ALDH1A2* locus, Pavan *et al*. found two mutations in patients with tetralogy of Fallot that change polar to non-polar residues at exon 4 of the gene. The alteration hindered tetramerization of retinaldehyde dehydrogenase, demonstrating concrete potential to disrupt the activity of the enzyme and contribute to the etiology of tetralogy of Fallot [7], the intracardiac lesion in our patient.

The association between retinoic acid and congenital diaphragmatic hernia is also strong. A variety of studies provide a foundation for the hypothesis that retinoids play a key role in the formation of the pleuroperitoneal fold, the embryonic structure that gives rise to the diaphragm [9]. As early as the 1940s, it was noted that 25% to 40% of offspring from vitamin A-deficient rats developed diaphragmatic hernia. The rate of diaphragmatic herniation in these rats decreased when vitamin A was introduced into the diet during mid-gestation [10]. Four different teratogens noted to induce congenital diaphragmatic hernia in rats exert their effect through dose-dependent inhibition of retinaldehyde dehydrogenase type 2 [11]. One such toxin, nitrofen, has been associated with a 50% incidence of congenital diaphragmatic hernia, and periodic co-morbid occurrence of cardiac malformation is observed in affected specimens [12]. Plasma levels of retinol and retinol-binding protein in babies with congenital diaphragmatic hernia have been found to be decreased by half of that of healthy neonates [13], lending clinical credence to the role of retinoids in the setting of this defect.

The longstanding suggested mechanism for the development of pentalogy of Cantrell has been the developmental failure of a segment of the lateral mesoderm at gestational age 14 to 18 days. As a consequence, the paired mesodermal folds of the upper abdomen do not migrate ventromedially and the transverse septum of the diaphragm does not develop [1]. It is plausible that altered conversion of retinaldehyde to retinoic acid secondary to *ALDH1A2* dysfunction could contribute to the developmental failure seen in Cantrell’s pentalogy. Analysis of complementary deoxyribonucleic acid (DNA) is required to determine if a maternal copy number gain is truly disruptive to retinoic acid concentrations in the developing fetus; however, it is plausible that such a gain is part of the mechanism underlying the pathology demonstrated in our patient with pentalogy of Cantrell.

## Conclusion

Given the robust evidence linking *ALDH1A2* to congenital diaphragmatic hernia, congenital heart disease in general, and tetralogy of Fallot in particular, it is a reasonable hypothesis that the gain in copy of the *ALDH1A2* gene in our patient and his mother played a role in the development of pentalogy of Cantrell. Further research targeting the *ALDH1A2* gene is warranted to ascertain the effect of copy number variants on the expression of retinaldehyde dehydrogenase type 2, and if subsequent alteration in maternal retinoic acid levels during development contribute to the etiology of pentalogy of Cantrell in children.

## Consent

Written informed consent was obtained from the patient’s guardian (mother) for publication of this case report and any accompanying images. A copy of the written consent is available for review by the Editor-in-Chief of this journal.

## Competing interests

The authors declare that they have no competing interests.

## Authors’ contributions

MS researched the subject matter and drafted the manuscript. JV and RC were coauthors who reviewed, edited and critically revised the manuscript. All authors approved the final manuscript.

## References

[B1] Cantrell JR Haller JA Ravitch MM A syndrome of congenital defects involving the abdominal wall, sternum, diaphragm, pericardium, and heart Surg Gynecol Obstet 1958 107 602 614 13592660

[B2] Lu XY Phung MT Shaw CA Pham K Neil SE Patel A Sahoo T Bacino CA Stakiewicz P Kang SH Lalani S Chinault AC Lupski JR Cheung SW Beaudet AL Genomic imbalances in neonates with birth defects: high detection rates by using chromosomal microarray analysis Pediatrics 2008 122 1310 1318 10.1542/peds.2008-0297 19047251 PMC2795566

[B3] Hartman RJ Rasmussen SA Botto LD Riehle-Colarusso T Martin CL Cragan JD Shin M Correa A The contribution of chromosomal abnormalities to congenital heart defects: a population-based study Pediatr Cardiol 2011 32 1147 1157 10.1007/s00246-011-0034-5 21728077

[B4] Thienpont B Mertens L de Ravel T Eyskens B Boshoff D Maas N Fryns JP Gewillig M Vermeesch JR Devriendt K Submicroscopic chromosomal imbalances detected by array-CGH are a frequent cause of congenital heart defects in selected patients Eur Heart J 2007 28 2778 2784 10.1093/eurheartj/ehl560 17384091

[B5] Mazzarella R Schlessinger D Pathological consequences of sequence duplications in the human genome Genome Res 1998 8 1007 1021 9799789 10.1101/gr.8.10.1007

[B6] Southard AE Edelmann LJ Gelb BD Role of copy number variants in structural birth defects Pediatrics 2012 129 755 763 10.1542/peds.2011-2337 22430448

[B7] Pavan M Ruiz VF Silva FA Sobreira TJ Cravo RM Vasconcelos M Marques LP Krieger JE Lopes AA Oliveira PS Pereira AC Xavier-Neto J ALDH1A2 (RALDH2) genetic variation in human congenital heart disease BMC Med Genet 2009 10 113 126 10.1186/1471-2350-10-113 19886994 PMC2779186

[B8] Zile MH Vitamin A-not for your eyes only: requirement for heart formation begins early in embryogenesis Nutrients 2010 2 532 550 10.3390/nu2050532 22254040 PMC3257662

[B9] Greer JJ Babiuk RP Thebaud B Etiology of congenital diaphragmatic hernia: the retinoid hypothesis Pediatr Res 2003 53 726 730 10.1203/01.PDR.0000062660.12769.E6 12621107

[B10] Andersen DH Effect of diet during pregnancy upon the incidence of congenital hereditary diaphragmatic hernia in the rat; failure to produce cystic fibrosis of the pancreas by maternal vitamin a deficiency Am J Pathol 1949 25 1 163 185 18122930 PMC1942780

[B11] Mey J Babiuk RP Clugston R Zhang W Greer JJ Retinal dehydrogenase-2 is inhibited by compounds that induce congenital diaphragmatic hernias in rodents Am J Pathol 2003 162 673 679 10.1016/S0002-9440(10)63861-8 12547725 PMC1851155

[B12] Costlow RD Manson JM The heart and diaphragm: target organs in the neonatal death induced by nitrofen (2,4-dichlorophenyl-p-nitrophenyl ether) Toxicology 1981 20 209 227 10.1016/0300-483X(81)90052-4 7256786

[B13] Major D Cadenas M Fournier L Leclerc S Lefebvre M Cloutier R Retinol status of newborn infants with congenital diaphragmatic hernia Pediatr Surg Int 1998 13 547 549 10.1007/s003830050399 9799371

